# Effect of roasting temperature on lipid and protein oxidation and amino acid residue side chain modification of beef patties†

**DOI:** 10.1039/d1ra03151a

**Published:** 2021-06-18

**Authors:** Chao Xia, Pingping Wen, Yaming Yuan, Xiaofan Yu, Yijing Chen, Huiqing Xu, Guiyou Cui, Jun Wang

**Affiliations:** College of Food Science and Engineering, Yangzhou University Yangzhou 225127 China xhq@yzu.edu.cn; Yangzhou Fangguang Food Co. Ltd. Yangzhou 225008 China

## Abstract

Beef is rich in nutrients and is one of the most important ingredients in the world. But in the process of cooking and heating, the nutrients of beef will change to varying degrees. How temperature affects the oxidation of lipids and proteins in beef, and the modification of amino acid residues is unclear. This study intended to heat beef at different roasting temperatures (150 °C, 190 °C, 230 °C, 270 °C, 310 °C), measure parameter including colour, peroxide value (PV), thiobarbituric acid-reactive substances (TBARS), thiol and carbonyl content, protein solubility, tryptophan and Schiff base content, protein molecular weight distribution and modification of amino acid residues to discussed the effects of different temperatures on the lipid and protein oxidation of beef patties, as well as the modification of amino acid residues. The results showed that the values of L* and b* increased with the temperature increased, and the values of a* decreased. With the increase of temperature, the lipid oxidation indexes PV and TBARS, Schiff base and carbonyl content also increased, and the thiol content and protein solubility decreased significantly (*p* < 0.001). SDS-PAGE showed that the band of myosin heavy chain (MHC, 220 kDa) was significantly degraded, while the band of actin (42 kDa) was still clearly visible. The analysis of UPLC-MS/MS results found that the aromatic amino acid residues in all samples were oxidized to a certain extent, especially tryptophan. Other oxidative modifications, including α-amiooadipic acid (AAA), hydroxyethyl lysine (CEL) and malondialdehyde (MDA), were only present in roasted samples and not in raw meat. The results suggested that lipid oxidation and protein oxidation were closely related to colour parameters. The oxidation of proteins and lipids was aggravated at higher temperature. Amino acid side chains were also modified at high temperature, and this change was particularly evident in aromatic amino acids. These results provided new insights for the oxidation of proteins and lipids of beef and the modification level of amino acid residues under high temperature conditions, which will help us to improve the cooking quality of meat foods.

## Abstract 2

The relative number of peptides modified by the amino acid residues of actin from raw beef patties and those cooked at different roasting temperatures.

## Graphical abstract

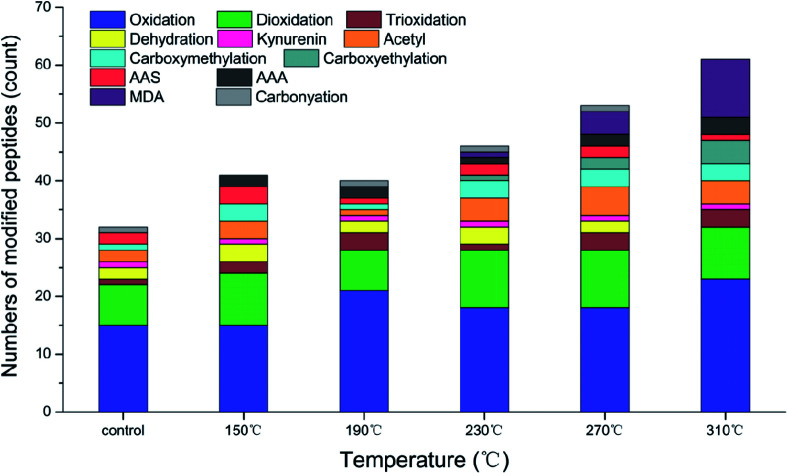

## Introduction

Meat can provide the human body with high-quality protein, essential amino acids, unsaturated fatty acids, minerals and vitamins, which are of great significance to human health and diet. In particular, red meat, such as beef, lamb and pork, is the main source of human protein.^1^ In domestic meat processing and maturation, traditional cooking methods include boiling, frying, steaming, microwave and roasting, which aim to remove pathogenic microorganisms and optimize sensory characteristics. Physical and chemical changes in meat caused by heating of cooking include water loss, protein aggregation and collagen fibre contraction, which change the colour, flavour, tenderness and nutritional values of the meat.^2,3^ Temperature and time are two key parameters in cooking, and their effect on the physical and chemical properties and eating quality of meat and consequently on consumer acceptance is a hot research topic. Many researchers reported that meat cooked at low temperature and for long periods of time had significantly higher tenderness, juiciness and overall acceptance compared with meat cooked by conventional heating methods.^4–6^ In particular, cooking temperature is closely related to protein denaturation and meat texture and thus has received increasing attention.

Protein and lipid oxidation are the main reasons for deterioration of meat quality. Continuous high temperature during cooking will increase the generation of reactive oxygen species (ROS), such as free radicals and non-radicals, which will strengthen the tendency of protein and lipid oxidation.^7^ Lipid oxidation products, such as hydroperoxide, malondialdehyde (MDA) and 4-hydroxy-2-nonenal (HNE), have cytotoxicity and mutagenic effect that can cause a variety of human diseases.^8^ Lipid oxidation is affected by many factors, and temperature has always been a topic of interest. Roldan *et al.*^9^ reported lower levels of thiobarbital active substances (TBARS) and hexanal at higher cooking temperature and longer time combinations. The formation of volatile secondary compounds seems to be related to temperature reached during cooking.^10^ In addition, the aldehyde moiety of MDA and HNE can indirectly cause protein oxidation by covalently binding to amino acid residues.

Cooking can increase the production of free radicals, and the attack mechanism of free radicals leads to the oxidation of amino acid side chains and protein backbones, which in turn leads to further fragmentation or cross-linking of proteins.^11^ All amino acid residues are easily attacked by ROS and undergo different types of oxidative modification because of the sensitivity of active sulphur atoms.^12^ The oxidative expression of protein in meat is mainly the loss of thiol groups, the increase of carbonyl groups, the formation of disulphide bonds and the loss of solubility under the heat induction of cooking. Meat protein will form abundant amino acid residue-modified products after cooking. For example, lysine forms α-aminoadipic semialdehyde (AAS) and advanced glycosylation end products (AGEs); AAS is further oxidized to a more stable α-aminoadipic acid (AAA). The modification of aromatic amino acids, such as tryptophan, produces kynurenine and 3-hydroxykynurenine. Carboxylysine is found in cooked and roasted lamb, and glucose-derived N-terminal Amadori compound can be found in roasted samples.^13,14^ Cooking produces higher level and greater diversity of amino acid modifications in mature fish fillets, especially in roasted and fried samples.^15^ In addition, higher cooking temperature and longer cooking times induce higher levels of oxidative damages and modification, although the degree varies from index to index.^16^

Roasting is a typical high-temperature cooking method that enhances the colour and flavour of meat through caramelization and Maillard reaction. Current research mainly focuses on the effect of low-temperature cooking for a long time or cooking time on meat. To our knowledge, the mechanism of beef protein–lipid oxidation and amino acid side chain modification on beef protein–lipid oxidation and amino acid side chain modification has not been elucidated. In order to evaluate the effects of different roasting temperatures on the cooking loss, colour, protein and lipid oxidation of beef, the temperature of 150 °C, 190 °C, 230 °C, and extreme high-temperature of 270 °C and 310 °C were selected to cook beef, and some parameters were tested to analyze the relationship between beef protein and lipid oxidation and the difference in amino acid modification under different conditions.

## Materials and methods

### Materials

Acrylamide, *N*,*N*-methylene bisacrylamide, glycine and tris base were purchased from Jintai Hongda Biotechnology Co., Ltd. (Beijing, China). 2-Thiobarbituric acid, dithiothreitol, thiourea, Coomassie Brilliant Blue R-250, sodium dodecyl sulphate (SDS), bromophenol blue and 2,4-dinitrophenylhydrazine were obtained from Sangon Biotech Co., Ltd. (Shanghai, China). *N*,*N*,*N*,*N*-tetramethylethylenediamine and β-mercaptoethanol were acquired from Macleans Biochemical Technology Co., Ltd. (Shanghai, China). Coomassie Brilliant Blue kit was provided by Nanjing Jiancheng Institute of Bioengineering (Jiangsu, China). 1,1,3,3-Tetraethoxypropane and chromatography grade formic acid were supplied by Sigma-Aldrich Co., Ltd. (MO, USA). Mass spectrometry-grade trypsin, acetonitrile and water were obtained from Thermo Fisher Scientific (MA, USA). All other chemical reagents were of analytical grade and purchased from Sinopharm Chemical Reagent Co., Ltd. (Shanghai, China).

### Preparation of samples

Beef was obtained from a local slaughterhouse (Yangzhou, China). At 48 h after slaughter, the longissimus dorsi was obtained from the carcasses of three beef cattle. A Seven2GO portable digital pH meter was used to measure pH in three different places in each muscle. The pH values of all muscles were within 5.5–5.7. After removing the visible fat and connective tissue on the surface, the muscle was cut into block samples (70 mm × 50 mm × 20 mm, length × width × height), vacuum packed, stored at −20 °C and quickly transported to the laboratory. The samples were slowly thawed at 4 °C for 12 h before the experiment, cut into 10 mm × 10 mm × 10 mm particles and processed into meat emulsion with a crusher. Each raw beef patty (50 ± 0.1 g) was formed with a Petri dish (6 × 1.5 cm) to ensure the uniformity of the samples. No condiments, such as oil and salt, were added to all patties to avoid other ingredients from interfering with the results. The patties were roasted in a electric oven (SM-523, Xinmai Machinery Co., Ltd., Wuxi, China). Before the start of each experiment, the oven was preheated to 150 °C, 190 °C, 230 °C, 270 °C or 310 °C and kept it for 30 min to ensure stable conditions. The patties were placed in the preheated oven and roasted for 10 min on each side. After cooling to room temperature, the samples were cut into small pieces, freeze-dried and stored at −80 °C prior to analysis. Roasting at different temperatures was carried out in three replicates. For each repetition, three beef patties were used for each treatment.

### Cooking loss

Cooking loss was calculated according to the method of Lonenzo *et al.*^17^ After cooking, the patties were naturally cooled to room temperature. The samples were weighed before and after roasting. The surface was dried with filter paper before weighing. Cooking loss was calculated as follows:
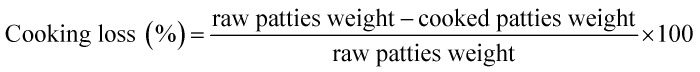


### pH

The pH was measured with the method of Li *et al.*^18^ with slight modifications. In brief, 10 g of minced meat was mixed with 90 mL of distilled water, homogenized at 12 000 rpm for 1 min by using an FJ200 high-speed dispersing homogenizer (Shanghai Specimen Model Factory, Shanghai, China) and then kept at 4 °C for 30 min. The suspension was measured using an FE20 pH meter (Mettler Toledo, Shanghai, China).

### Colour measurement

Colour was measured using a portable colourimeter (Chroma Meter CR-400, Minolta Co., Ramsey, Japan) on the surface of three beef patties cooled to room temperature in the same series. The L* (lightness), a* (redness) and b* (yellowness) values of the samples were obtained using the internal built-in illuminator D65 light source. The colourimeter was calibrated using a white standard tile (L* = 83.4, a* = 0.3185, b* = 0.3249) before the measurement.

### Peroxide value (PV)

The PV was evaluated with the method of Mi *et al.*^19^ with slight modifications. In brief, 0.5 g of a lyophilized sample was homogenized in 10 mL of cold chloroform–methanol (1 : 1) at 12 000 rpm for 15 s. Samples were then added with 3 mL of 0.5% sodium chloride solution and centrifuged at 1800 rpm for 10 min at 4 °C. The bottom chloroform layer (2.5 mL) was collected by a glass syringe and transferred to a glass tube. An aliquot of the 1 mL chloroform layer was added to 50 μL of ferrous chloride and 50 μL of ammonium thiocyanate and then vortexed to mix for 10 s. The resulting solution was incubated at room temperature for 20 min. Absorbance of the solution was recorded at 500 nm. Ferric chloride solution (10 μg mL^−1^) was used to construct a standard curve. Results were expressed as milliequivalents of hydrogen peroxide per kilogram of dry weight.

### Thiobarbituric acid-reactive substances (TBARS)

The TBARS values of beef patties were measured with the method of Witte *et al.*^20^ with minor modifications. In brief, 1 g of a lyophilized sample was homogenized in 15 mL of trichloroacetic acid (TCA) reagent (7.5% TCA, 0.1% EDTA) in an ice bath. The resulting homogenate was filtered by Whatman qualitative filter paper, and the filtrate was collected. The filtrate (2 mL) was mixed with 2 mL of 0.02 M TBA solution and heated in a boiling water bath at 100 °C for 40 min. After cooling, the absorbance of the solution was recorded at 532 nm. TBARS values was calculated from the standard curve of 1,1,3,3-tetraethoxypropane (TEP). Results were expressed as milligrams of malondialdehyde per kilogram of dry weight.

### Protein solubility

Total protein solubility and sarcoplasmic protein solubility were measured according to the method described by Joo *et al.*^21^ with slight modifications. Total protein was extracted from 0.5 g of samples with 10 mL of ice-cold 0.1 M potassium phosphate butter (pH 7.2, 1.1 M potassium iodide) and used for total protein solubility determination. After the samples were chopped, it was homogenized three times at 12 000 rpm in an ice bath for 30 s each time and then shaken on a shaker at 4 °C for 12 h. The homogenate was centrifuged at 1500 rpm for 20 min at 4 °C. The protein concentration of the supernatant was determined using Biuret method.^22^ Sarcoplasmic protein was extracted from 0.5 g of samples with 0.025 M ice-cold phosphate buffer (pH 7.2) and subjected to the same processing and determination procedure as above. The solubility of myofibrillar protein was calculated by subtracting the solubility of sarcoplasmic protein from the total protein solubility. Results were expressed as milligrams of soluble protein per gram of muscle (mg per g).

### Free thiols and total thiols

The content of free thiols and total thiols were determined according to the method of Ellman^23^ by using 5,5′-dithiobis (2-nitrobenzoic acid) (DTNB) with some modifications. In brief, 1 g of samples was homogenized in 20 mL of 50 mM phosphate buffer (pH 7.0) at 3000 rpm for 60 s. The mixture was centrifuged at 2000 rpm for 15 min at 4 °C. Two aliquots of 0.5 mL supernatant were collected. One aliquot was mixed with 50 mM phosphate buffer (pH 7.0) containing 0.6 M NaCl and 10 mM EDTA. The other aliquot was mixed with 50 mM phosphate buffer (pH 7.0) containing 8 M urea, 0.6 M NaCl and 10 mM EDTA. Both aliquots were used for determination of free thiol and total thiol. After 5 min, the diluted supernatant was added to 0.02 mL of 0.1% DTNB and incubated in the dark at 40 °C for 30 min. The absorbance of the solution was read at 412 nm. The content of free thiols and total thiols was calculated with a molar extinction coefficient of 13.6 mM^−1^ cm^−1^. Results were expressed as nmol of thiols per milligram of protein.

### Carbonyl content

The level of protein carbonyl groups was determined using 2,4-dinitrophenylhydrazine (DNPH) derivatization method described by Levine.^24^ In brief, 1 g of samples was homogenized for 60 s in 10 mL of 20 mM phosphate buffer containing 0.6 M NaCl. Two aliquots of the homogenate were collected, and 1 mL of ice-cold 20% TCA was added and used to precipitate the protein. The mixture was centrifuged at 12 000 rpm for 5 min at 4 °C, and the supernatant was discarded. After centrifugation, one pellet was added with 1 mL of 2 M HCl containing 0.2% DNPH, and the other pellet was added with 1 mL of 2 M HCl as a blank control and incubated for 1 h at room temperature. After the reaction, the samples were precipitated by 1 mL of 40% TCA and centrifuged (12 000 rpm, 5 min, 4 °C). Samples were washed with 1 mL of ethanol: ethyl acetate (v/v) three times to remove excess DNPH. The pellet was dissolved in 1.5 mL of 20 mM phosphate buffer (pH 6.5) containing 6 M guanidine hydrochloride and centrifuged at 5000 rpm for 2 min to remove insoluble fragments. The absorbance of the supernatant was recorded at 370 nm. Bovine serum albumin was used as the standard, and protein concentration was measured at 280 nm. Carbonyl content was calculated using the molar absorptivity of 22.0 mM^−1^ cm^−1^ and expressed as nmol per mg of protein carbonyl.

### Fluorescence measurements of tryptophan and Schiff base structures (SB)

The natural fluorescence of tryptophan and the fluorescence emission of the Schiff base (SB) structure of the protein oxidation product were evaluated using F-320 fluorescence spectroscopy (F-320, Gangdong Technology, Tianjin, China) according to the method of Estevez *et al.*^25^ In brief, 1 g of samples was homogenized in 20 mL of sodium phosphate buffer (pH 6.5) containing 0.6 M NaCl for 30 s and then filtered with gauze. The homogenate (1 mL) was re-dissolved in 20 mL of 20 mM sodium phosphate buffer and then distributed in 4 mL quartz spectrofluorometer cell. The emission spectrum of tryptophan was recorded from 300 nm to 400 nm with excitation wavelength at 283 nm. The emission spectrum of Schiff base was recorded from 400 nm to 500 nm with excitation wavelength at 350 nm. In both measurements, the excitation and emission slit widths were set at 10 nm, and the data were collected at a speed of 500 nm min^−1^.

### SDS-PAGE

Muscle protein was extracted using the method described by Kim *et al.*^26^ Samples were immersed in liquid nitrogen and ground into powder. In brief, 1 g of meat powder was added to 16 mL of 8 M urea, 2 M thiourea, 2% (w/v) dithiothreitol (DTT), 2% (w/v) CHAPS and 1% (v/v) biological bacteria. Supernatant was obtained after the samples was homogenized and centrifuged. The Bradford kit was used to determine the protein concentration of the solution, and the concentration was adjusted to 4 mg mL^−1^. The urea–thiourea extract was mixed with 5× sample buffer (60 mM Tris–HCl (pH 6.8), 25% glycerol, 2% SDS and 0.1% bromophenol blue) with or without 14 mM β-mercaptoethanol in 3 : 1 (v/v) ratio and heated in a water bath at 80 °C for 10 min. Approximately 20 μg of the protein was run on top of SDS polyacrylamide gel (10% T, 5% C) in a VE-180 Mini Vertical Electrophoresis Cell (Tanon Science & Technology Co., Ltd. Shanghai, China) with a constant voltage of 120 V for 90 min. The gel was stained in Coomassie Brilliant Blue R-250 solution for 2 h and then destained in methanol–acetic acid solution (10% methanol, 10% acetic acid and 80% distilled water) until the protein bands were clearly visible. The gel was photographed on the R-2500 gel imaging system.

### In-gel trypsin digestion

The gel bands of all samples were cut from the gel lane near 42 kDa (length × width, 5 mm × 1 mm) and then placed in a centrifuge tube. The gel slice was decolorized in 50 mM ammonium bicarbonate and 30% ACN. After 15 min of sonication, the supernatant was discarded and washed repeatedly until it became colourless. The samples was reduced by adding 100 μL of 100 mM ammonium bicarbonate solution containing 10 mM DTT and incubated at 58 °C for 45 min. Samples were alkylated in 100 μL of 100 mM IAA and reacted at room temperature for 40 min. After the gel slice was washed with 100 μL of 100 mM ammonium bicarbonate, it was added with 90 μL of 100% ACN and incubated for 10 min. The gel was digested in 20 μL of trypsin solution (10 ng μL^−1^ in 25 mM ammonium bicarbonate) at 37 °C for 15 h. After the reaction, 200 μL of the peptide extract (50% ACN, 0.1% TFA) was added immediately and reacted for 30 min. The product was transferred to a new EP tube, and the extraction was repeated twice. The extract was freeze dried and stored at −80 °C until analysis.

### UPLC-MS/MS conditions and data analysis

The lyophilized samples were dissolved in 20 μL of 0.1% formic acid solution and centrifuged at 14 000 rpm for 15 min. Supernatant (15 μL) was collected and analyzed by ultra-high-performance liquid phase chromatography coupled with Orbitrap Fusio Lumo Tribrid mass spectrometer (Thermo Fisher Scientific, USA). The mass spectrometer was composed of a quadrupole mass filter, a linear ion trap and an Orbitrap mass analyzer. The samples were loaded on a C18 column (Acclaim PepMap100, 2 cm × 100 μm, C18, 100 Å; Thermo Fisher Scientific, USA), and then separated by an analytical column (EASY-Spray, 15 cm × 75 μm, C18, 3 μm, 100 Å; Thermo Fisher Scientific, USA). The flow rate was set to 600 nL min^−1^, and the injection volume was 5 μL. The mobile phase was composed of eluent A (0.1% formic acid in water) and eluent B (0.1% formic acid in 80% acetonitrile). The gradient elution program was optimized as follows: 0–2 min (94% A, 6% B); 2–10 min (91% A, 9% B); 10–50 min (87% A, 13% B); 50–70 min (74% A, 26% B); 70–71 min (62% A, 38% B); 71–78 min (100% B). Positive ion detection mode was used for mass spectrometry (MS) analysis at a spray voltage of 2.3 kV. The primary resolution was 120 000, and the AGC was set to 5 G^5^. The scanning range was set to 300–1400 *m*/*z*. Twenty ions with the strongest signal were selected for secondary mass spectrometry analysis, and the rejection time was set to 18 s. The ion trap fast scan was used, the AGC was set to 5000 and the separation window was 1.6 *m*/*z*. The MAXQUANT search engine was used for analysis of mass spectrometer data. Data were retrieved using the *Bos taurus* peptide database from refseq at the National Centre for Bioinformatics NCBI (https://www.ncbi.nlm.nih.gov/). The following search parameters were used for data matching: (1) trypsin as the proteolytic enzyme; (2) precursor ion tolerance: 10 ppm; (3) fragment ion tolerance: 0.8 Da; (4) two missed cleavages were allowed; (5) the significance threshold was *p* < 0.05. The search modification types were shown in ESI.†

### Statistical analysis

All experiments were performed in triplicate, and data were expressed as mean ± standard deviation of three replicates. The effect of roasting temperature on cooking loss, pH, PV, TBARS, total carbonyls, protein solubility, free thiol groups and total thiol groups were evaluated by one-way analysis of variance (ANOVA) by using SPSS 19.0 software (SPSS Inc., Chicago, IL, USA). Duncan's multiple range test was used to compare significant difference between means, and the difference was considered significant at *p* < 0.05.

## Results and discussion

### Cooking loss and pH

Cooking loss is a common phenomenon during heat treatment of food and is affected by factors, such as heating temperature, heating time and nature of raw material. Heating can cause thermal denaturation of muscle protein, including denaturation of myosin and contraction of myofibrils, thereby causing water discharge and increasing cooking loss.^27^ The cooking loss of beef patties is shown in Table 1. In the present study, cooking loss of all samples increased significantly (*p* < 0.001). With increasing temperature, cooking loss increased significantly from 15.1% at 150 °C to 43.2% at 310 °C (*p* < 0.05); in particular, cooking loss increased sharply from 19.2% to 32.7% between 190 °C and 230 °C (*p* < 0.05). Consistent with our results, the findings of Bıyıklı *et al.*^28^ indicated that cooking temperatures (65 °C, 70 °C and 75 °C) had a significant effect on the cooking loss of turkey patties. As the roasting temperature increased, pH values also increased significantly (*p* < 0.05), ranging from 5.56 to 5.64. The difference between 230 °C and 310 °C was not significant (*p* > 0.05). Vasanthi *et al.*^29^ also reported that the pH of buffalo meat cooked at 100 °C was higher than that of buffalo meat cooked at 80 °C and 90 °C.

**Table 1:** Cooking loss, pH and colour parameters of beef patties roasted at different temperatures^a^

Index	Temperature					
	Control	150 °C	190 °C	230 °C	270 °C	310 °C
Cooking loss (%)	∼	15.1 ± 0.4^e^	19.2 ± 0.7^d^	32.7 ± 1.5^c^	37.5 ± 0.7^b^	43.2 ± 0.9^a^
pH	5.48 ± 0.02^c^	5.56 ± 0.03^b^	5.62 ± 0.01^a^	5.65 ± 0.03^a^	5.68 ± 0.01^a^	5.64 ± 0.08^a^
L* (lightness)	36.17 ± 1.04^b^	41.57 ± 1.14^a^	41.79 ± 0.33^a^	31.68 ± 1.84^c^	30.31 ± 0.75^c^	26.65 ± 0.38^d^
a* (redness)	18.63 ± 0.10^a^	18.69 ± 1.91^a^	10.49 ± 0.52^b^	9.65 ± 0.61^b^	8.77 ± 0.68^b^	9.74 ± 0.53^b^
b* (yellowness)	11.19 ± 0.15^b^	14.54 ± 0.45^a^	14.64 ± 0.78^a^	11.72 ± 0.67^b^	11.75 ± 0.50^b^	11.32 ± 0.56^b^

a Mean values denoted with different letters in superscripts in the same row are statistically significantly different (*p* < 0.05).

### Colour parameters

Colour is one of the main quality attributes of meat and is considered to directly affect consumer acceptance and selectivity.^30^ The colour measurement values (L*, a* and b*) of beef patties changed with fluctuations in the roasting temperature (Table 1). The change in colour of beef patties may be caused by the interaction of lipid and protein oxidation during roasting. The beef patties roasted at 150 °C and 190 °C showed significantly higher L* values than raw meat (*p* < 0.05), which may be due to denaturation and aggregation of sarcoplasmic protein and myofibrillar protein, thereby increasing light scattering.^31^ Lorenzo *et al.*^17^ also reported that roasting significantly increased L* values of foal meat. However, the significantly reduced L* values at higher roasting temperatures (230 °C, 270 °C and 310 °C) can be attributed to the decrease in moisture content caused by the increase in roasting temperature. Red (a* values) was the direct change in the colour of beef patties during roasting, and the degree of myoglobin denaturation was closely related to the a* values.^32^ In addition, lipid oxidation would increase the number of free radicals present in meat, leading to an increase in the rate of myoglobin oxidation.^33^ When the temperature was increased from 150 °C to 190 °C, a* values decreased significantly (*p* < 0.05) and then remained stable. When the temperature was between 150 °C and 190 °C, yellowness (b* values) of samples increased significantly (*p* < 0.05); however, no significant changes (*p* > 0.05) were found at higher temperatures (230 °C, 270 °C and 310 °C). Heat treatment promotes the denaturation of myoglobin and the formation of heat-denatured methemoglobin, which may contribute to the increase in b* values.^34^

In this study, lipid oxidation led to an increase in L* and b* values. Lipid oxidation indicators (PV and TBARS) and colour measurements (L* and b*) showed similar trends (Tables 1 and 2). Colour analysis showed that beef patties roasted at 150 °C and 190 °C had higher L* and b* values, but their a* values decreased with increasing temperature.

**Table 2:** Effect of different roasting temperatures on lipid oxidation, protein oxidation and protein solubility of beef patties^a^

Indexes	Temperature					
	Control	150 °C	190 °C	230 °C	270 °C	310 °C
**Lipid oxidation**						
PV (meq peroxide per kg dry weight)	1.16 ± 0.07^b^	1.69 ± 0.01^a^	2.17 ± 0.03^a^	1.82 ± 0.16^a^	1.46 ± 0.07^b^	1.36 ± 0.07^b^
TBARS (mg MDA per kg dry weight)	1.37 ± 0.13^b^	1.71 ± 0.23^a^	1.66 ± 0.29^a^	0.97 ± 0.07^b^	0.94 ± 0.04^b^	0.88 ± 0.08^b^

**Protein oxidation**						
Total thiol (nmol per mg protein)	83.90 ± 7.79^a^	43.83 ± 0.39^b^	21.86 ± 1.92^c^	20.36 ± 0.61^c^	15.35 ± 1.28^d^	7.97 ± 0.85^e^
Free thiol (nmol per mg protein)	48.19 ± 7.65^a^	28.94 ± 1.30^b^	19.02 ± 3.13^c^	20.16 ± 0.51^c^	13.87 ± 0.66^d^	6.60 ± 1.46^e^
Total carbonyls (nmol per mg protein)	0.53 ± 0.04^d^	2.71 ± 0.49^c^	8.61 ± 0.39^b^	7.98 ± 0.09^ab^	11.14 ± 0.41^a^	12.00 ± 0.66^a^

**Protein solubility**						
Total protein solubility (mg g^−1^)	69.25 ± 3.85^a^	19.48 ± 1.82^b^	7.93 ± 0.39^c^	8.00 ± 0.60^c^	7.11 ± 0.67^c^	4.43 ± 0.75^d^
Sarcoplasmic protein solubility (mg g^−1^)	13.85 ± 0.77^a^	4.90 ± 0.28^b^	1.69 ± 0.07^c^	1.64 ± 0.21^c^	1.51 ± 0.17^c^	1.46 ± 0.08^c^
Myofibrillar protein solubility (mg g^−1^)	55.40 ± 3.08^a^	14.58 ± 1.80^b^	6.24 ± 0.44^c^	6.35 ± 0.53^c^	5.60 ± 0.57^c^	2.97 ± 0.79^d^

a Mean values denoted with different letters in superscripts in the same row are statistically significantly different (*p* < 0.05).

### Lipid oxidation

Lipid oxidation is the main factor that influences the internal quality, external colour and smell of food. Lipid oxidation is the complex reaction of unsaturated fatty acids with molecular oxygen through a free radical mechanism and is accompanied with the formation of a series of oxidation products, such as aldehydes, alcohols, ketones, acids and hydrocarbons.^8^ Hydroperoxide is considered the primary oxidation product of lipids and is expressed as the level of PV. The initial PV of the control was 1.16 meq kg^−1^. With increasing roasting temperature, PV increased rapidly (*p* < 0.05), reaching the peak value (2.17 meq kg^−1^) at 190 °C. The PV of samples decreased significantly at 270 °C (*p* < 0.05) as the temperature was further increased. This finding may be due to the instability and easy decomposition of hydroperoxide and that high temperatures decrease its content.^35^ TBARS values represents the content of malondialdehyde produced during the secondary oxidation of lipids. Consistent with the PV, TBARS values showed an upward and then downward trend. The TBARS values of beef patties roasted at temperature above 230 °C decreased significantly (*p* < 0.05) and was lower than that of the control samples (*p* > 0.05); this finding could be attributed to the binding of aldehyde compounds produced during the secondary lipid oxidation to the protein. Jin *et al.*^36^ reported that the PV and TBARS values of bacon during drying–salting and drying–curing treatments decreased with increasing temperature. Roldan *et al.*^9^ found that the TBARS values of meat was reduced after a long period of high-temperature heating.

### Protein solubility

Protein solubility is an important indicator of the degree of denaturation and aggregation during protein oxidation. This parameter is closely related to many protein functional properties that affect the quality of meat products, such as water holding capacity, emulsification ability and foaming properties.^9^ The results of the solubility of total proteins (TP), myofibrillar proteins (MP) and sarcoplasmic proteins (SP) at different temperatures are shown in Table 2. Compared with the control, all heat-treated samples had very low solubility of the three proteins (*p* < 0.001). This finding indicated that the oxidation and denaturation of protein were enhanced with increasing temperature, and the binding capacity of protein and water was reduced through hydrophobic interaction. Taşkıran *et al.*^37^ and Wazir *et al.*^38^ also reported the decrease in muscle protein solubility after heat treatment. The solubility of beef MP was significantly decreased at 150 °C to 190 °C and 270 °C to 310 °C (*p* < 0.05); however, no significant difference was found from 190 °C to 270 °C (*p* > 0.05). MP is approximately composed of about 43% myosin and 22% actin.^39^ Myosin was very unstable at the beginning of roasting and was easily decomposed at high temperatures, resulting in the decrease in MP solubility. Actin has good thermal stability, so MP solubility remains stable at 190–270 °C (*p* > 0.05). In the current study, the solubility change in TP was found to be the same as that of MP possibly because MP accounts for 55–60% of TP. No significant difference in the solubility of beef SP was detected at 190–310 °C (*p* > 0.05), indicating the complete denaturation of SP. According to reports, most sarcoplasmic proteins accumulate at 40–60 °C, and some can be extended to 90 °C. Similarly, changes in core temperature had no effect on the solubility of water-soluble and salt-soluble proteins during cooking of large-mouth bass.^40^

### Protein oxidation

Protein oxidation is defined as the covalent modification and oxidative stress of protein caused by direct interaction with reactive oxygen species (ROS) or indirect interaction with secondary by-products, such as modification and scission of amino acid side chains and protein–protein cross-linkages.^41^ The inevitable heat treatment in cooking is believed to trigger the generation of ROS, the reduction of enzyme antioxidant activity and the modification of aromatic residues. The progress of protein oxidation is often accompanied by loss of protein thiols. The reduction of total thiols and free thiols during the production of beef patties is shown in Table 2. The concentrations of total thiol and free thiol groups in fresh beef samples were 83.9 and 48.2 nmol mg^−1^ protein, respectively. The thiol groups was significantly reduced (*p* < 0.001) with increasing temperature, indicating that all samples had undergone protein oxidation. After roasting, the minimum content of total thiol and free thiol were 8.0 and 6.0 nmol per mg protein, respectively, which decreased by 90.46% and 87.55% compared with those of the control and appeared in the samples cooked at 310 °C. The level of thiol remained stable without significant loss (*p* > 0.05) at 190 °C and 230 °C.

In addition to the loss of thiols, protein oxidation can be reflected by the formation of carbonyl compounds. Protein carbonylation occurs through physical and chemical reactions caused by high temperatures, such as the release of iron and the formation of hydroperoxides. The variation in the total carbonyl of beef cooked at different temperatures is shown in Table 2. With increasing temperature, the carbonyl content of each treatment group were 5.11, 16.25, 15.06, 21.02 and 22.64 times of fresh meat (0.53 nmol per mg protein). A significant negative correlation was found between temperature and carbonyl content (*p* < 0.001). However, no significant difference in carbonyl content (*p* > 0.05) was detected at 190–230 °C and 270–310 °C. Within a certain temperature range, the carbonyl content will not increase or decrease significantly. This result was consistent with the observation of Wang *et al.*^40^ on the carbonyl content of fish after different cooking temperatures. Therefore, the changes of thiol groups and carbonyl groups confirm each other in the process of increasing temperature. Previous studies showed that the thiol level of dry-cured chicken protein was decreased and the carbonyl level increased with increasing drying temperature at the same NaCl concentration.^42^ Similarly, Bhaskar *et al.*^16^ reported the effect of heat treatment on the oxidation of pork protein also obtained the same trend.

### Fluorescence measurements of tryptophan and Schiff base structures

**Fig. 1:**
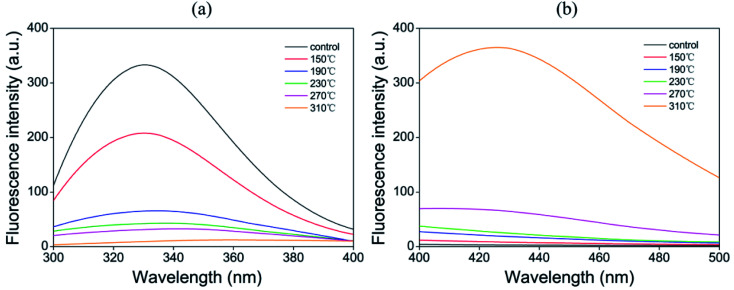
Effect of different temperatures on the fluorescence spectra of control raw meat and cooked sample extracts. (a) Tryptophan fluorescence intensity after excitation at 283 nm and (b) Schiff base fluorescence intensity after excitation at 350 nm.

Tryptophan is an aromatic amino acid that is very sensitive to oxidation and is used as one of the markers of protein oxidation loss. The oxidative modification of tryptophan is mainly manifested as the loss of natural fluorescence intensity, which can be evaluated by fluorescence spectroscopy.^16^ As shown in Fig. 1, the fluorescence intensity of tryptophan was significantly reduced among beef patty samples of all treatments. The oxidation of tryptophan is due to the release of catalytic iron in the heme molecule and the inactivation of enzymes induced by high temperature during cooking, leading to the destruction of muscle tissues and the cleavage of hydroperoxide.^43^ In the present study, the roasting temperature was inversely proportional to the fluorescence intensity of tryptophan; the reaction rate of the process was accelerated with increasing temperature, thereby showing higher fluorescence loss of tryptophan. Silva *et al.*^44^ also found that the tryptophan content of the roasted samples was significantly reduced at higher cooking temperatures (200 °C/15 min) compared with those of the samples in the other cooking treatments. In addition, the tryptophan levels of samples at 190 °C, 230 °C, 270 °C and 310 °C were further reduced, although the difference was small. This finding indicated that the formation of crosslinks was promoted by oxidation at higher temperatures (above 190 °C).

The adjacent amino groups of basic amino acids (mainly lysine) of proteins and the aldehyde part from lipid oxidation products (mainly α-aminoadipic semialdehyde) undergo an addition reaction to form Schiff bases (SB).^43^ The effect of different temperatures on the fluorescence of Schiff bases varied (Fig. 1). The fluorescence intensity increased slightly among the samples treated at 150 °C, 190 °C and 230 °C compared with that of raw meat. At 150 °C to 230 °C, the difference in temperature had little effect on the formation of Schiff bases. According to reports, the formation of SB seems to be related to heating time. Traore *et al.*^7^ found that the SB fluorescence of pork increased significantly with prolonged cooking time.

By contrast, the Schiff base fluorescence of beef patties at 310 °C increased significantly due to the more active lipid oxidation at high temperatures; moreover, the oxidation products reacted with protein amino groups to release more Schiff base compounds.

### SDS-PAGE

**Fig. 2:**
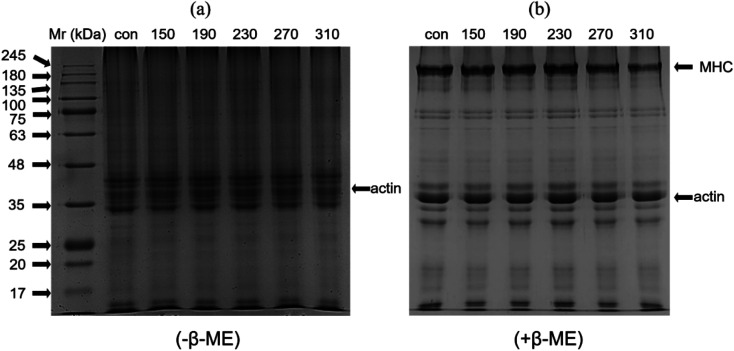
Effect of different roasting temperatures (150 °C, 190 °C, 230 °C, 270 °C and 310 °C) on the SDS-PAGE patterns of raw and cooked beef patties. Samples were run under non-reducing (a, without β-ME) and reducing (b, with β-ME) SDS-PAGE conditions.

The changes of beef protein with the increase of temperature under non-reducing and reducing conditions is shown in Fig. 2. The SDS-PAGE profile showed that the band patterns of the roasted samples at different temperatures and the control had significant differences. Under non-reducing conditions without β-mercaptoethanol (β-ME), the band strength of myosin heavy chain (MHC, 220 kDa), actin (actin, 42 kDa) and myosin light chain (MLCs, 16–28 kDa) were decreased with increasing roasting temperature. The most obvious was that the intensity of the MHC band was gradually weakened until it disappeared completely, probably because the MHC was not stable to heat, and high temperature can cause its strong degradation and production of low molecular weight protein and MLCs.^7^

After the treatment of β-mercaptoethanol, most of the bands of MHC, myosin and actin were restored, indicating that the cross-linking of proteins during heating occurred *via* disulphide bonds. The temperature changes produced by cooking can induce the accumulation of proteins in the muscles through non-covalent and covalent interactions, leading to the oxidation of myosin and the formation of high-molecular polymers and in turn causing protein–protein cross-linking and presence on the top of the gel.^45^ These results indicated that temperature was the main factor that causes protein aggregation and degradation. The higher the temperature was, the more significant the degree and speed of degradation will be.

The actin band intensity of the roasted samples was gradually reduced under non-reducing and reducing conditions compared with that of the control. Actin has good thermal stability.^46^ Wan *et al.*^47^ found that actin band was slightly faded but was still clearly visible after heating for 180 min at 100 °C.

### Peptide profiles of trypsin-treated protein

Muscles were constantly exposed to various forms of reactive oxygen species (ROS), such as free radicals, non-free radicals and reactive aldehydes and ketones, which are produced in the biological metabolic system. Cooking can increase the production of free radicals.^11^ Free radical-mediated oxidative modification mainly occurs on the side chains of amino acids, such as the hydroxylation of aromatic residues, the formation of carbonylation and glycosylation products and the interaction between protein and lipid oxidation products.^41^

**Fig. 3:**
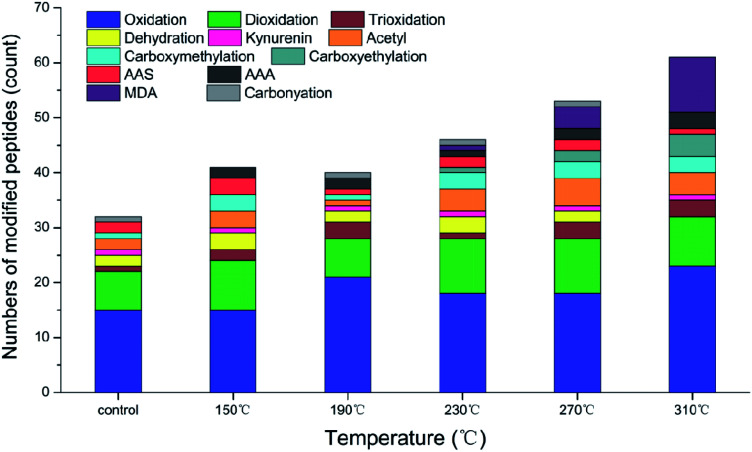
Relative number of peptides modified by the amino acid residues of actin from raw beef patties and those cooked at different roasting temperatures.

Actin has abundant peptides and good thermal stability. Compared with those of myosin and sarcoplasmic protein, the peptide sequence of actin is the main site for various modifications of amino acid residues during processing.^11,14^ The modified peptides and the corresponding amino acid residues in the roasted samples are shown in Table 3. As shown in Fig. 3, the high temperature of roasting led to a continuous increase in the number of modified amino acid peptides due to a series of physical and chemical changes as a result of the high temperature of the environment. The number of modified peptides decreased in the samples cooked at 190 °C, and some modified and oxidation products such as γ-glutamic semialdehyde (GGS) and α-aminoadipic acid (AAA) were only present in the samples at specific roasting temperatures.

**Table 3:** Heat-induced modification of amino acid residues in beef patties at different temperatures (150 °C, 190 °C, 230 °C, 270 °C and 310 °C).^a^

Residue	Sequence	Modification	Treatment					
			Control	150 °C	190 °C	230 °C	270 °C	310 °C
Cysteine	**C̲**PETLFQPSFIGMESAGIHETTYNSIMKC	Oxidation			✓		✓	
		Dioxidation			✓			
Proline	C**P̲**ETLFQPSFIGMESAGIHETTYNSIMKC	Dioxidation			✓			
Phenylalanine	CPETL**F̲**QPSFIGMESAGIHETTYNSIMKC	Oxidation			✓	✓	✓	✓
Serine	CPETLFQP**S̲**FIGMESAGIHETTYNSIMKC	Dehydration		✓		✓		
Phenylalanine	CPETLFQPS**F̲**IGMESAGIHETTYNSIMKC	Oxidation	✓	✓	✓	✓	✓	✓
		Dioxidation	✓	✓		✓	✓	✓
		Trioxidation					✓	
Methionine	CPETLFQPSFIG**M̲**ESAGIHETTYNSIMKC	Oxidation	✓	✓	✓	✓	✓	✓
		Dioxidation				✓	✓	✓
Tyrosine	CPETLFQPSFIGMESAGIHETT**Y̲**NSIMKC	Oxidation	✓	✓	✓	✓	✓	✓
		Dioxidation	✓			✓	✓	✓
Methionine	CPETLFQPSFIGMESAGIHETTYNSI**M̲**KC	Oxidation	✓	✓	✓	✓	✓	✓
		Dioxidation	✓	✓		✓	✓	✓
Lysine	CPETLFQPSFIGMESAGIHETTYNSIM**K̲**C	Acetyl		✓				✓
		Carboxymethylation		✓		✓	✓	✓
Serine	TTGIVLD**S̲**GDGVTHNVPIYEGYALPHAIMRLDLAGR	Dehydration	✓	✓	✓	✓	✓	✓
Tyrosine	TTGIVLDSGDGVTHNVPI**Y̲**EGYALPHAIMRLDLAGR	Trioxidation						✓
Proline	TTGIVLDSGDGVTHNVPIYEGYAL**P̲**HAIMRLDLAGR	Oxidation	✓	✓	✓	✓	✓	✓
		Dioxidation		✓			✓	✓
Arginine	TTGIVLDSGDGVTHNVPIYEGYALPHAIM**R̲**LDLAGR	MDA				✓		✓
Tryptophan	I**W̲**HHTFYNELR	Oxidation	✓	✓	✓	✓	✓	✓
		Dioxidation	✓	✓	✓	✓	✓	✓
		Trioxidation	✓	✓	✓		✓	
		Kynurenin	✓	✓	✓	✓	✓	✓
Histidine	IW**H̲**HTFYNELR	Trioxidation				✓	✓	✓
Histidine	IWH**H̲**TFYNELR	Oxidation				✓		✓
Arginine	IWHHTFYNEL**R̲**	MDA						✓
Histidine	**H̲**QGVMVGMGQKDSYVGDEAQSK	Oxidation			✓		✓	✓
		Dioxidation	✓		✓	✓	✓	✓
Methionine	HQGV**M̲**VGMGQKDSYVGDEAQSKR	Oxidation	✓	✓	✓	✓	✓	✓
		Dioxidation	✓		✓	✓		✓
Lysine	HQGVMVGMGQ**K̲**DSYVGDEAQSKR	Acetyl					✓	
		Carboxymethylation				✓		
Tyrosine	HQGVMVGMGQKDS**Y̲**VGDEAQSKR	Oxidation	✓		✓		✓	✓
		Dioxidation				✓		
Lysine	HQGVMVGMGQKDSYVGDEAQS**K̲**R	Acetyl					✓	✓
Cysteine	L**C̲**YVALDFENEMATAASSSSLEK	Oxidation	✓	✓	✓	✓		✓
		Dioxidation			✓			
Tyrosine	LC**Y̲**VALDFENEMATAASSSSLEK	Oxidation	✓	✓	✓	✓	✓	✓
Methionine	LCYVALDFENE**M̲**ATAASSSSLEK	Oxidation	✓	✓	✓	✓	✓	✓
		Dioxidation		✓			✓	
Lysine	LCYVALDFENEMATAASSSSLE**K̲**	Acetyl				✓	✓	✓
		Carboxymethylation					✓	✓
Proline	VA**P̲**EEHPTLLTEAPLNPKANR	Oxidation						✓
Proline	VAPEEH**P̲**TLLTEAPLNPKANR	Oxidation			✓	✓		
Histidine	VAPEE**H̲**PTLLTEAPLNPKANR	Oxidation	✓	✓	✓		✓	✓
		Dioxidation		✓	✓	✓	✓	
		Trioxidation			✓			✓
Proline	VAPEEH**P̲**TLLTEAPLNPKANR	Oxidation		✓	✓	✓	✓	✓
Lysine	VAPEEHPTLLTEAPLNP**K̲**ANR	Carboxyethylation						✓
		MDA						✓
Arginine	VAPEEHPTLLTEAPLNPKAN**R̲**	MDA						✓
Lysine	**K̲**DLYANNVMSGGTTMYPGIADR	Acetyl	✓	✓	✓	✓	✓	✓
		Carboxymethylation	✓	✓	✓	✓	✓	✓
		Carboxyethylation					✓	✓
		AAS		✓				
		Carbonylation	✓		✓	✓	✓	
Tyrosine	KDL**Y̲**ANNVMSGGTTMYPGIADR	Oxidation	✓	✓	✓	✓	✓	✓
Methionine	KDLYANNV**M̲**SGGTTMYPGIADR	Oxidation	✓	✓	✓	✓	✓	✓
		Dioxidation	✓	✓	✓	✓	✓	✓
Serine	KDLYANNVM**S̲**GGTTMYPGIADR	Dehydration	✓	✓	✓	✓	✓	✓
Methionine	KDLYANNVMSGGTT**M̲**YPGIADR	Oxidation	✓	✓	✓	✓	✓	✓
Proline	EITALA**P̲**STMKIKIIAPPERK	Oxidation			✓	✓		
Lysine	GILTL**K̲**YPIEHGIITNWDDMEK	Carboxyethylation					✓	
Proline	GILTLKY**P̲**IEHGIITNWDDMEK	Dioxidation		✓				
Histidine	GILTLKYPIE**H̲**GIITNWDDMEK	Dioxidation		✓				
Phenylalanine	AV**F̲**PSIVGRPR	Oxidation						✓
Proline	AVF**P̲**SIVGRPR	Oxidation						✓
Tryptophan	YPIEHGIITN**W̲**DDMEKIWHHTFYNELR	Trioxidation		✓				
Lysine	YPIEHGIITNWDDME**K̲**IWHHTFYNELR	Acetyl	✓			✓	✓	
		Carboxymethylation		✓				
		Carboxyethylation				✓		✓
Lysine	E**K̲**LCYVALDFENEMATAASSSSLEK	Acetyl		✓		✓		
		Carboxyethylation						✓
Lysine	RGILTL**K̲**YPIEHGIVTNWDDMEKIWHHTFYNELR	AAA		✓	✓		✓	✓
		AAA						✓
Lysine	RGILTLKYPIEHGIVTNWDDME**K̲**IWHHTFYNELR	Oxidation						✓
Methionine	DLTDYL**M̲**KILTER	AAA		✓	✓	✓	✓	✓
Lysine	KVKSE**K̲**ADKLLK	AAS	✓	✓	✓	✓	✓	
Lysine	**K̲**GEISELLVGSPSIR	AAS	✓	✓		✓	✓	✓
Lysine	SNSLVSSFPME**K̲**R	MDA						✓
Arginine	P**R̲**HQGVMVGMGQK	MDA					✓	
Arginine	F**R̲**CPETLFQPSFIGMESAGIHETTYNSIMK	MDA					✓	✓
Arginine	ILTE**R̲**GYSFVTTAER	MDA					✓	✓
Arginine	AGFAGDDAP**R̲**AVFPSIVGR	MDA					✓	✓
Arginine	GYSFVTTAE**R̲**EIVR	MDA						✓
Arginine	SYELPDGQVITIGNE**R̲**FR	MDA						✓

a ✓ means that the corresponding modification type was detected in the sample at the corresponding temperature. Blank means not detected. The bold and underlined letters in the peptide sequence indicate the modified amino acid residues.

Aromatic amino acid residues, including tryptophan, tyrosine and phenylalanine, are the main targets of protein attack by ROS. High temperature may cause structural damages and residue modification.^46^ Aromatic amino acid residues were modified into various oxidation products and the main sites in actin were within the following six peptides: CPETLFQPSFIGMESAGIHETTYNSIMKC, IWHHTFYNELR, HQGVMVGMGQKDSYVGDEAQSK, LCYVALDFENEMATAASSSSLEK, VAPEEHPTLLTEAPLNPKANR and KDLYANNVM (Table 3). In this study, a series of different oxidative modifications were identified in three aromatic amino acid residues. Oxidation, di-oxidation and tri-oxidation of tryptophan were found in raw meat and various roasted samples. At the same time, the oxidation of tryptophan was also manifested in the loss of fluorescence intensity (Fig. 1). Kynurenine and 3-hydroxykynurenine, intermediate products of tryptophan metabolism indicated further oxidation of tryptophan. The same level of kynurenine and the absence of 3-hydroxykynurenine were found with increasing roasting temperature, indicating that temperature may have no effect on the production of kynurenine. The tri-oxidation of phenylalanine and tyrosine are mainly concentrated in samples at 270 °C and 310 °C. Gatellier *et al.*^48^ reported the thermal stability of three aromatic amino acids (tryptophan, phenylalanine and tyrosine) in beef was significantly affected at three cooking temperatures (60 °C, 100 °C and 140 °C). In addition, amino acids containing active sulphur atoms such as cysteine, proline, methionine and histidine were oxidized to varying degrees.

The physical and chemical changes caused by high temperature during cooking can facilitate the formation of protein carbonyls from specific amino acid residues on the amino acid side chains. This phenomenon is an irreversible and non-enzymatic modification. AAS is the oxidative deamination product of lysine residues through the metal-catalyzed reaction. GGS is the oxidation product of proline and arginine through Maillard reaction.^49^ Although the level of AAS was higher than the control at 150 °C, it decreased with increasing temperature. This finding may be due to the further oxidation of AAS and lipid peroxides at high temperature, resulting in a stable final oxidation product of advanced lysine: α-amiooadipic acid (AAA). At the same time, this results explained the phenomenon that the PV of beef patties decreased with increasing roasting temperature. AAA was only found in the roasted samples, and the content reached the maximum in the samples at 310 °C. Utrera *et al.*^43^ reported similar results. The carbonylation modification of lysine was not observed in the samples cooked at 310 °C. The formed carbonylation product may undergo more drastic changes at higher temperature conditions until it cannot be recognized.

Currently recognized lipid oxidation modification biomarkers are malondialdehyde (MDA) and 4-hydroxy-2-nonenal (HNE), which are the secondary oxidation products of unsaturated fatty acids and n-6 polyunsaturated fatty acids, respectively. The MDA modification of arginine was identified on many peptides modified above 230 °C; in particular, the number of modifications increased twofold at 270 °C and 310 °C. Lysine was also modified on a peptide (VAPEEHPTLLTEAPLNPKANR). High levels of heme iron in red meat, such as beef and lamb, are released and converted into non-heme iron. The oxidation of myoglobin is related to lipid oxidation during heat induction.^50^ In our experiments, this process seems to be accelerated with increasing temperature and the reduction in a* values of beef patties due to myoglobin oxidation also confirms this result. The TBARS value of beef patties were inconsistent with the result of MDA modification, the reason may be that the measurement object of TBARS was beef patties, but the modification of MDA was identified on the peptide of actin.

In the glycosylation of protein side chains, lysine residues easily react with reducing sugars to form advanced glycosylation end products (AGEs), such as carboxymethyllysine (CML) and carboxyethyllysine (CEL).^51^ More carboxymethylation of lysine was found in roasted samples than in uncooked meat, but the increase in temperature did not affect the carboxymethylation level of lysine in mature samples, except for the samples at 190 °C. Carboxyethylation of lysine was only detected in beef patties cooked at higher temperatures (230 °C, 270 °C and 310 °C) because the rate of Maillard reaction was accelerated with increasing temperature, resulting in the formation of new Maillard chemicals. At the same time, the Maillard reaction of two peptides of actin, namely, TTGIVLDSGDGVTHNVPIYEGYALPHAIMRLDLAGR and KDLYANNVMSGGTTMYPGIADR, is also affected by the general dehydration of serine caused by heat treatment.

In addition to the above modifications, protein lysine acetylation is one of the important protein post-translational modifications during cell regulation of energy metabolism.^52^Table 3 shows the modified acetylation of lysine residues on the actin peptides of raw beef. The acetylation modification of muscles after slaughter continues to be affected by the regulation of energy metabolism. Similarly, Zhou *et al.*^53^ reported consistent results. Different roasting temperatures will cause differences in the degree of acetylation of beef protein, which is manifested as a high level of acetylation at 230 °C to 310 °C. As a dynamically reversible process, the role and mechanism of acetylation modification in the cooking and processing of meat products still need further research.

### Correlation analysis

Correlation analysis was completed to establish relationships between some of parameters in this study (Table 4). The results shows that positive significant correlations were obtained between TBARS values of beef patties and colour parameters L*, a* and b* values (*p* < 0.01), the content of free thiol and total thiol (*p* < 0.05), and there were negative significant correlations between TBARS values and cooking loss, pH and carbonyl content (*p* < 0.01). In addition, a positive significant correlation was obtained between PV values and b* values (*p* < 0.05), and there was a negative significant correlation between total thiol content and solubility of three proteins (*p* < 0.05). Solubility of three proteins had a positive significant correlation with free thiol and total thiol (*p* < 0.01), and a negative significant correlation with carbonyl content (*p* < 0.01). Therefore, during the heating process, colour parameters, lipid oxidation, protein oxidation and protein solubility of beef patties were closely related, and decrease of protein solubility was also related to degree of protein oxidation.

**Table 4:** Correlation matrix between indexes at different roasting temperatures^a^

	CL	pH	L*	a*	b*	PV	TBARS	T-SH	F-SH	Carbonyls	TR-S	SP-S	MP-S
CL	1.000												
pH	0.849**	1.000											
L*	−0.728**	−0.467	1.000										
a*	−0.841**	−0.847**	0.564*	1.000									
b*	−0.264	−0.072	0.810**	0.240	1.000								
PV	0.230	0.468	0.342	−0.364	0.560*	1.000							
TBARS	−0.738**	−0.617**	0.884**	0.644**	0.724**	0.181	1.000						
T-SH	−0.920**	−0.855**	0.435	0.854**	−0.073	−0.471*	0.491*	1.000					
F-SH	−0.923**	−0.799**	0.497*	0.817**	−0.022	−0.352	0.506*	0.977**	1.000				
Carbonyls	0.942**	0.829**	−0.696**	−0.887**	−0.268	0.232	−0.674**	−0.881**	−0.893**	1.000			
TR-S	−0.839**	−0.821**	0.254	0.758**	−0.253	−0.603**	0.342	0.967**	0.913**	−0.763**	1.000		
SP-S	−0.870**	−0.848**	0.313	0.795**	−0.186	−0.571*	0.405	0.976**	0.922**	−0.800**	0.995**	1.000	
MP-S	−0.830**	−0.814**	0.239	0.748**	−0.269	−0.610**	0.326	0.963**	0.910**	−0.753**	1.000**	0.992**	1.000

a CL, cooking loss; T-SH, total thiol content; F-SH, free thiol content; TR-S, total protein solubility; SP-S, sarcoplasmic protein solubility; MP-S, myofibrillar protein solubility. * indicates significant correlation (*p* < 0.05); ** indicates extremely significant correlation (*p* < 0.01).

## Conclusions

Roasting temperature has an important influence on the physical and chemical properties as well as lipid and protein oxidation of beef patties. High temperature significantly increase the cooking loss, increase the pH and lead to different colour values. The samples at 150 °C and 190 °C have higher L* and b* values, and the degradation of myoglobin causes the continuous decrease in the a* values. In addition, the interaction of protein and lipid leads to the increase in the PV and TBARS values first and then decrease due to the formation of Schiff base substances. Indicators of protein oxidation include the increase in the carbonyl content, the loss of thiol groups and the decrease in the fluorescence intensity of tryptophan. With increasing roasting temperature, the level of protein oxidation also increases. Specific amino acid residue modifications are evaluated through proteomics techniques. Amino acid residue modifications showed greater diversity and higher levels at higher temperatures. The oxidation of aromatic amino acid residues, particularly lysine residues, forms AAS, AAA and advanced glycosylation end products, such as CML and CEL. AAA and CEL are characterized in the roasted samples. The lipid oxidation product MDA was identified in samples at higher temperatures (230 °C, 270 °C and 310 °C). Therefore, during cooking, temperature was of great significance to the acceptance and nutritional values of meat.

## Author contributions

These authors contributed equally to this work.

## Conflicts of interest

The authors declare that they have no conflicts of interest.

## Supplementary Material

RA-011-D1RA03151A-s001
