# Supplementary material for: Effect of roasting temperature on lipid and protein oxidation and amino acid residue side chain modification of beef patties
Source: RSC Adv. 2021 Jun 18;11(35):21629–41. doi: 10.1039/d1ra03151a (PMC9034085; doi:10.1039/d1ra03151a)
Supplement: RA-011-D1RA03151A-s001 [file RA-011-D1RA03151A-s001.pdf]

Electronic Supplementary Material (ESI) for RSC Advances.
This journal is © The Royal Society of Chemistry 2021

Supplementary information:

List of modifications caused by heating

	Modification type	Target residue	Position	Element	Molecular mass（Da）
1	Oxidation	CMFHPWY	Any	O(1)	15.99
2	Dioxidation	CMFHPWY	Any	O(2)	31.99
3	Trioxidation	CFHWY	Any	O(3)	47.98
4	Dehydration	S	Any	H(-2)O(-1)	-18.01
5	Dehydroalanine	Y	Any	C(-6)H(-6)O(-1)	-94.04
6	Kynurenin	W	Any	C(-1)O(1)	3.99
7	Hydroxykynurenine	W	Any	C(-1)O(2)	19.99
8	Acetyl	K	Any	C(2)H(2)O	42.01
9	Carboxymethylation	K	Any	C(2)H(2)O(2)	58.01
10	Carboxyethylation	K	Any	C(3)H(4)O(2)	72.02
11	α-Aminoadipic semialdehyde (AAS)	K	Any	H(-3)N(-1)O	-1.03
12	α-Amiooadipic acid (AAA)	K	Any	H(-3)N(-1)O(2)	14.96
13	Malondialdehyde (MDA)	KR	Any	C(3)H(2)O	54.01
14	4-Hydroxy-2-nonenal (HNE)	CHKR	Any	C(9)H(16)O(2)	156.12
15	Carbonylation	KR	Any	H(-2)O(-1)	18.01
